# Medicine as a Business

**Published:** 1900-12

**Authors:** 


### "MEDICINE AS A BUSINESS."

DR. G. FRANK LYDSTON, of Chicago, is a versatile writer, and he has the happy faculty of saying things which sink deep down into the memory and stick there. He recently wrote a work on genito-urinary diseases which is quite the most satisfactory article of its kind which has come along in some time. And now he has issued a little pamphlet which, with all due respect to the vast army of genito-urinary men, amateurs and professionals, is apt to do much more good generally than all the text-books in the medical business. Text books teach a man how to be a doctor, but Dr.

Lydston's little pamphlet will teach doctors how to be men, and we all know some who need educational advantages. They can get a post-graduate course in Lydston's pamphlet and it will be cheap. This pamphlet is a noisy little publication. It does a whole lot of talking and it talks straight and hard, and it is known as "Medicine as a Business."

We can well imagine the warmth of the discussion which followed the address on which the pamphlet is based. Dr. Lydston, himself, speaks of it. Concerning the financial rewards of the profession, the writer tells of the tradesman who, while owing his family physician a goodly amount, sends his goods C. O. D., or, if an account is permitted, the bill comes booming along the first of the month followed by a collector on the 15th. This is too true; too sadly true. Look over the judgment rolls of Erie County and see the physicians' names; search in vain for the name of a judgment whose account is for medical services! The greatest fault lies at the door of the profession itself. Doctors do not send out bills often, and when they do they let them go for what they are worth and seldom if ever follow them up with a collector. In this respect there is a growing sentiment in Buffalo for a betterment of financial affairs, and a number of physicians now never mail bills at all, but merely send a collector out every month to present them. An excellent illustration of one class of people spoken of by Dr. Lydston—the "dead-beat" fraternity—lives in Buffalo. He has been ill six times in two years and has had eight different physicians, not one of whom has received a cent for his services. And he gets the best, too. The "free" patient comes in for his share of lashing at Dr. Lydston's hands, as does the grasping hospital and the dispensary. In Buffalo, this feature obtains to a large extent. One young woman who needed an operation made arrangements with a surgeon to perform it for $50. She had the money and the day was fixed. In the meantime she secured a "poor order," went to a hospital and was operated upon. It did not cost her a cent for anything. The grasping after business tends to make dispensaries and hospitals overlook investigation and this is one thing which Dr. Lydston complains of bitterly. It would do a world of good to distribute these pamphlets broadcast. It would tend to elevate the medical profession higher than all the codes of ethics in the minds of any of our ethical societies or practitioners. Send to the *Riverton Press* and get a copy of the pamphlet, follow its advice and a great change will come over your fortunes and your friends! You'll have more of both.

Now that a general election has been held and the Republican party has been retained in power, it is time something were done for the medical department of the Army. As it is now constituted it is wholly inadequate. There are too few surgeons for the number of men in the regular and volunteer regiments and, although Gen. Sternberg is doing his very best to keep the army well supplied with medical attendance, he is hampered by restrictive laws and arbitrary rulings by the comptroller of the treasury.

The employment of surgeons by contract is at best a make-shift and although they have done remarkably good service and have been simply invaluable, there is no inducement for them to go into foreign service, or remain there after the expiration of their contracts. The contract surgeons should be commissioned and given all the rights and privileges of other officers. The welfare of the army is in the hands of the administration, and the army bill should be pushed through early in the coming session.

---

THE Texas *Medical News*, published at Austin, devotes considerable space in its October edition to an account of the hurricane that enveloped Galveston, September 8, 1900, with such terrible destruction of life and property. The article is graphically written and copiously illustrated with half-tone engravings, which depict the awful ravages of a storm that swept the Island City with the besom of destruction. A sympathising world has offered its assistance to a suffering and sorrowing people, already struggling manfully to repair the almost immeasurable damage done by wind and water in a few hours.

---

THE Michigan health administration is proverbially among the best. The high regard in which it is held by well-informed people not only in the state of Michigan but in other states and countries, is found in the preface to the first bulletin of the American Congress of Tuberculosis held in New York wherein the secretary of that organisation, who is also the president of the Medico-legal Association, says relative to the restriction of tuberculosis: "that it is a proper subject for legislation, for the use of all proper means to prevent or arrest its spread or increase, is now very generally conceded and the Michigan legislation and the statistics from that state show that similar legislation in other states and still more radical measures might be of great public good, in arresting the alarming spread of this scourge of the human race."

